# A review of the evidence for the effectiveness of primary prevention interventions for Hepatitis C among injecting drug users

**DOI:** 10.1186/1477-7517-3-27

**Published:** 2006-09-06

**Authors:** Nat MJ Wright, Charlotte NE Tompkins

**Affiliations:** 1 Her Majesty's Prison Leeds, Leeds, UK; 2 Leeds West Primary Care Trust, Leeds, UK

## Abstract

**Background:**

Hepatitis C (HCV) prevalence is most common amongst injecting drug users where up to 98% of the population can be infected despite a low prevalence of HIV. This review considers the evidence for the effectiveness of primary prevention interventions to reduce incidence or prevalence of hepatitis C.

**Methods:**

Systematic review of the major electronic medical databases: Medline, EMBASE, PsycINFO, CINAHL and the Cochrane Library (Evidence Based Health). Either intervention or observational studies were included if they described an intervention targeting injecting drug using populations with the outcome to reduce either the prevalence or incidence of hepatitis C infection.

**Results:**

18 papers were included in the final review from 1007 abstracts. Needle exchange programmes reduce the prevalence of HCV though prevalence remains high. Similarly the effectiveness of methadone maintenance treatment is only marginally effective at reducing HCV incidence. There is limited evidence evaluating either the effectiveness of behavioural interventions, bleach disinfectants, or drug consumption rooms.

**Conclusion:**

Primary prevention interventions have led to a reduction in HIV incidence, have been less effective at reducing HCV incidence. Global prevalence of HCV remains disturbingly high in injecting drug users. A robust response to the global health problem of HCV will require provision of new interventions. Behavioural interventions; distribution of bleach disinfectant; other injecting paraphernalia alongside sterile needle distribution; and evaluation of drug consumption rooms merit further expansion internationally and research activity to contribute to the emerging evidence base. Whilst the prevalence of HCV remains high, nevertheless many current interventions aimed at primary HCV prevention have been shown to be cost-effective due to their significant positive impact upon prevalence of HIV.

## Background

Hepatitis C (HCV) is a blood borne virus (BBV) with potentially devastating hepatic complications [1]. While approximately 20% of acutely infected people will clear the virus and recover, up to 80% will develop chronic hepatitis C [2]. The World Health Organization (WHO) estimates that 3% of the world's population is infected [3] and hepatitis C has been declared a global public health problem. Nucleotide sequence analysis has highlighted six HCV genotypes which can be further categorized according to subtypes [4]. Differing genotypes are distributed differently by geographical region and route of infection, and have differing sensitivity to anti-viral treatment regimes [5]. In Japan, North America and Western Europe the majority of genotypes are numbers 1, 2 and 3, whereas genotype 4 is more prevalent in the Middle East and in North and Central Africa. Types 5 and 6 have been identified in South Africa and South East Asia, respectively [6].

While a number of risk factors have been identified, intravenous drug use is the major mode of HCV transmission [2,7]. Other transmission risk factors include receiving a blood transfusion or blood products before the availability of heat-treated factors in the mid 1980s in the UK, using non-sterilized equipment in dental, surgical, skin piercing and tattooing procedures, clinical injuries from dental or surgical procedures or needle stick injuries [8-10], vertical transmission (materno-fetal) and sexual spread [1].

A systematic review of HCV prevalence or incidence data for injecting drug users (IDUs) in European Union (EU) countries identified 98 studies [11]. Prevalence ranged from 30% to 95% among males, 48% to 94% among females and 33% to 98% among those of unspecified gender. This wide range in prevalence is confirmed by the European Monitoring Centre for Drugs and Drug Addiction (EMCDDA) [12,13], and concurs with a systematic review of seroprevalence of HCV markers among intravenous drug users (IVDUs) in western Europe [14]. Associations between increasing age, increasing duration of IDU or imprisonment and anti-HCV seropositivity were described. However, caution should be exercised in considering solely the results of prevalence studies when exploring risk factors for anti-HCV seroconversion. In addition to describing associations and not causal relationships, different countries differ in the data sources used to collect prevalence data. Additionally, in some situations, biochemical tests may underestimate prevalence. There are also warnings about comparing prevalence data with previous versions to follow changes over time, as inclusion of sources may vary according to data availability [13]. However prevalence data is not solely a marker of primary prevention, which is the process of preventing disease transmission. It is also a marker of secondary prevention, the process of eradicating the disease in those with established infection.

Therefore, to further understand the epidemiology of HCV so as to explore the effectiveness of primary prevention interventions, the international studies of anti-HCV incidence must be considered. The range of reported incidence of anti-HCV seroconversion is from 11 to 29 per 100 person-years [10,15-19]. Independent risk factors for HCV seroconversion include a history of imprisonment, a history of needle or other paraphernalia sharing and polydrug use, in particular using heroin and cocaine together [10,15,16,19]. While some incidence studies report younger age being an independent risk factor, others report older age [19]. However, the latter is strongly confounded with the duration of the injecting career and this is arguably a greater independent risk factor than age for anti-HCV seroconversion. The difficulty of adequately controlling for confounders of age was highlighted in a review of prevalence studies which described a linear positive relationship between increasing age and prevalence of anti-HCV-RNA in anti-HCV positive injecting populations [14]. The commentators offered possible explanations that HCV infection is more likely to resolve at a younger age, the natural history of the disease is characterized by frequent initial long periods of undetectable viral load levels, and age increases the risk of continuing exposure and re-infection. Similarly, there is no concordance between incidence studies as to whether gender is an independent risk factor, as some report a higher incidence in males [16], and others in females [17]. It is therefore possible that gender is confounded with other independent variables.

## Methods

### Search strategy

A full copy of the search strategy is available from the authors upon request. Briefly the following databases were searched: Medline, EMBASE (1980 to 2003 week 23), PsycINFO (1872 to April week 2 2003), CINAHL (1982 to March week 4 2003) and the Cochrane Library (Evidence Based Health) using search terms related to "drugs" "drug use" and "hepatitis C". Additionally, the index pages of the last five years publications of selected relevant, high-impact journals were searched by hand. The internet was also searched using key terms relating to hepatitis C and injecting drug use and reference lists of relevant papers were scanned. The search was not limited solely to publications in the English language (though not all identified papers were translated as many once retrieved were opinion pieces or descriptive studies). Possibility of publication bias was reduced by speaking with experts regarding relevant unpublished grey literature.

### Study selection

The protocol for selection criteria was informed by acknowledged historical political difficulties in obtaining research funding for experimental research in the field of reducing harm amongst drug users [20]. Either intervention or observational studies were included in the review if they described a primary prevention intervention targeting injecting drug using populations with the outcome to reduce either the prevalence or incidence of hepatitis C infection. Abstracts identified were reviewed by two researchers independently against agreed inclusion and exclusion criteria. Any discrepancy was resolved by discussion.

Descriptive studies, qualitative studies, editorials and opinion pieces were excluded from the review. Due to space constraints interventions targeting the general population (e.g. screening of blood products or prevention of vertical transmission) whilst alluded to in the original synthesis [21] are not included in this review. Quality of the studies was based on a checklist of criteria to include: clear case definition of anti-HCV positivity (type of biochemical test used); location (city, country, number and type of treatment settings); years of recruitment (and total duration of recruitment); number of participants (and breakdown by age, gender, ethnicity, sexual orientation, type of drug used, mean length of illicit drug use, employment status, housing status); percentage of those identified recruited into study; percentage follow-up of participants.

The checklist devised specifically for randomised controlled trials covered: a clear description of the randomisation process and whether open, single blind, or double blind; clear description of the concealment process; steps taken to avoid contamination; steps taken to ensure independence of data analysis; use of intention-to-treat analysis. The checklist for quasi-experimental or case-control studies covered: whether baseline data were reported; potential for selection bias described and accounted for in the analysis; potential for confounders described and accounted for either by multivariate analysis or stratification; steps taken to ensure independence of data analysis. Finally, the checklist devised specifically for observational cohort studies covered: whether probabilistic sampling methods were used to select participants; use of a control group; potential confounders described with an attempt made to quantify the effect either by multivariate statistical analysis or stratification; potential for loss to follow-up bias described and accounted for in the analysis (as a minimum description of any difference in baseline demographics between those followed up and those lost to follow-up).

## Results

**Figure 1:**
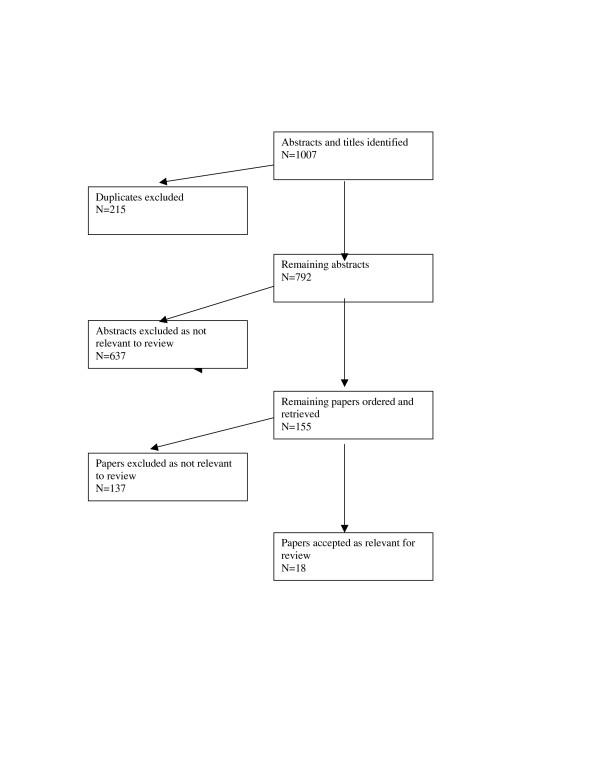
Papers Identified in the Systematic Review.

The review process identified 1007 abstracts. 155 full text papers were retrieved of which 18 met the inclusion criteria (see figure 1). The included papers were categorised according to type of intervention. 11 papers were categorised according to the theme of "needle exchange", 3 according to the theme "opiate replacement therapy", 1 according to the intervention of "bleach disinfectant", and 3 according to "expanded harm reduction" (where the harm reduction interventions of needle exchange, methadone maintenance, safer injecting advice or the effect of counsellors/therapists was not evaluated independently) and none to drug consumption rooms. No intervention studies were identified and of the observational studies identified, the intervention of needle exchange was the most evaluated. It also appeared to be the intervention that had been most contentious when first introduced. For these reasons a précis of the historical debates as they related to the topic of HIV transmission and also cost effectiveness evaluations are reported below. This is in addition to the studies observing their effectiveness as a primary prevention intervention. No intervention studies assessing the impact of harm reduction interventions at reducing hepatitis C in prisons were identified in the search.

As no intervention studies were identified it was not appropriate to conduct a meta-analysis. Rather the results are reported in the form of a narrative systematic review. Such a narrative format has been described as appropriate in reporting the results of observational studies identified through a process of systematic review [22]. The terms "antibodies to HCV", "HCV antibodies", "anti-HCV positive" and "anti-HCV seroconversion" are common terms used in the literature to describe a positive antibody response to HCV infection. However, not all those who are anti-HCV positive are viremic. Active viral replication as evidenced by the presence of serum viral RNA means that the person is a carrier of HCV. Such a state is referred to as anti-HCV-RNA positive. Successive generations of tests have led to an improved sensitivity and specificity of testing [23]. Currently anti-HCV seropositivity is assessed by third-generation enzyme-linked immunosorbent assay (ELISA) test. Unless specifically stated otherwise, where anti-HCV seropositivity is reported, a third-generation ELISA test was used for diagnosis.

### Can needle exchange programmes reduce prevalence of HCV?

The evaluation of the effectiveness of needle exchange programmes (NEPs) at reducing the risk of blood-borne viruses has been limited for several reasons. These include political legitimacy (which has been variable between different countries) as historically NEPs have been a contentious subject; difficulty quantifying the direct effect of NEPs as often there is an interaction with other factors causing a reduction (e.g. provision of bleach or counselling); or the effect of secondary exchange [20]. Evaluation has also been hampered as it is deemed unethical to evaluate using randomised control trial methodology. The limitations of observational research have been the difficulty in mitigating against selection bias of the most high risk users into NEPs. This limitation has on occasions fuelled the debate concerning the possibility of needle exchanges actually causing an increase in blood borne virus transmission.

An example of this was the contentious debate following the outbreak of HIV in Vancouver, Canada in 1994 [24]. The rapid rise in HIV prevalence was preceded by the introduction of an NEP in 1989. Prior to the outbreak Vancouver had a low HIV prevalence rate and it was assumed that this was due to the effectiveness of the NEP. The outbreak led to several observational studies which sought to explore a possible causal link between the NEP and the HIV outbreak. An initial outbreak investigation in 1995 found an independent association between needle sharing, and social determinants (such as unstable housing) and HIV seroconversion [25]. This led to a prospective cohort study of 1006 IDUs. Whilst the limited number of HIV seroconverters precluded a formal early statistical analysis, multivariate analysis of baseline data documented an independent association between HIV-positive serostatus and frequent (>once per week) NEP attendance. NEPs were thus criticised for promoting unsafe injecting drug use behaviour (or at the very least condoning injecting drug use). It was postulated that the NEP could act as a focus for forming social networks conducive to the initiation into unsafe injecting practice. Political ramifications were highlighted in the USA where the results were interpreted as evidence of a causal link between NEP use and HIV seroconversion leading to a continued ban on the use of federal funds to support NEPs [26-28]. However longitudinal analysis of HIV incidence amongst a sample of 694 subjects was reported in 1999 [28]. Univariate analysis of the data could have led one to postulate a causal link between the NEP and HIV seroconversion as cumulative incidence was significantly elevated in frequent attenders at the NEP. However frequent attenders were younger and more likely to report: unstable housing and hotel living; the downtown eastside part of the city as their primary injecting site; frequent cocaine injection; sex trade involvement; injecting in "shooting galleries"; or incarceration within the previous six months. Multivariate analysis to account for these confounders demonstrated that there was no independent causal link between NEP attendance and HIV seroconversion.

Within such a contentious international context, a series of large observational studies conducted in Scotland in the mid-1990s compared prevalence of anti-HCV for the periods before during and after introduction of NEPs. The supporting data and full results are presented in a summary of relevant studies [see Additional File 1] [29-32]. Results showed a statistically significant reduction in anti-HCV prevalence in the early 1990s (shortly after the introduction of NEPs). Reduction was greatest in the under 25s. However, evaluation in the late 1990s showed that the declining trend in overall prevalence did not continue. There was only a reduction for those aged over 25. The authors concluded that the incidence of HCV decreased during the 1990s, but remained high. Such findings are confirmed by an Australian prevalence study showing a reduction in anti-HCV incidence from 63% in 1995 to 51% in 1996 to 50% in 1997 [33], a Swedish cohort study [34] and a Swiss longitudinal and cross-sectional survey (including serological testing) [35,36]. The latter reported a reduction in anti-HCV prevalence after 1991 (when both needles and syringes were available) compared to 1988–1990 (when needles but not syringes were available) compared to before needle and syringe exchange in 1987. Two American studies failed to find a causal link between NEPs and HCV incidence. One case control study showed non-use of NEPs to be associated with a seven-fold greater risk of anti-HCV seroconversion [37]. The other, a prospective cohort study, showed a statistically non-significant increase in HCV with NEP use [38]. One Canadian study had insufficient power to determine a reducing trend in HCV incidence over the study period [17].

Whilst not studying the outcome of anti-HCV incidence, two large observational studies conducted in the United States demonstrate that the introduction of NEPs leads to a self-reported reduction in sharing when associated with an increase in distribution. Such increase in distribution does not lead to an increase in injecting drug use or a switch from non-injecting to injecting [39,40].

### Cost-effectiveness of needle exchange programmes

One of the most comprehensive reports on the cost effectiveness of NEPs was published by the Commonwealth Department of Health and Ageing of Australia in 2003 [41]. Employing ecological study methodology, changes in HCV and HIV prevalence were compared in cities that had NEPs with those that did not. There were 190 calendar years of HCV seroprevalence data from 101 cities. Pre-NEP introduction HCV prevalence rates of 75% or 50% corresponded to a 1.5% or 2% decline in HCV prevalence per annum. The cost-effectiveness of NEPs is optimized by the combined effect of reduction in HIV and reduction in HCV. The financial return on government investment in NEPs regarding the impact on HIV and HCV combined was calculated at a lifetime saving to costs of treatment of $3 653AUD million in treatment costs. A total gain of 170 279 Quality Adjusted Life Years (QALYs) were also calculated due to avoiding HCV and HIV. These findings concurred with American research that conducted a random mixing statistical model using sensitivity analysis to quantify the cost-effectiveness of NEPs in reducing the incidence of HCV [42], concluding that NEPs need to be integrated as part of broader interventions to reduce the population prevalence of HCV and thus maximize cost-effectiveness.

### Effect of opiate replacement therapy on HCV seroconversion

While buprenorphine and methadone are the two most common agents used for opiate replacement therapy, no studies evaluating the effectiveness of buprenorphine could be located. As regards methadone maintenance therapy, whilst it has been successful in reducing the incidence of HIV, the evidence for its effectiveness in reducing HCV incidence is less convincing [see Additional File 1] [18,43-47]. Indeed, an Italian nested case control study evaluated the impact of MMT on 746 injecting heroin users [45]. 263 IDUs were HCV negative at baseline and 106 (40.3%) underwent re-testing. Total follow up time was 73.4 person years, during which time 21 individuals seroconverted, an incidence rate of 28.6 per 100 person years (95% CI 17.8–43.4). The adjusted odds ratio for "lack of methadone treatment" (in the six months prior to testing) was of borderline significance (2.9, 95% CI 0.9–9.7).

Such equivocal conclusions were also the findings of a prospective cohort study assessing causal associations between retention in methadone treatment and HCV in 716 IDUs in Seattle, USA [46]. Participants were categorised into either left treatment, disrupted treatment or continued treatment. There was a marked difference in reducing or stopping injection between the treatment status groups and the primary outcome variables measured the incidence of HCV or HBV over the study period. Multivariate analysis showed a non-statistically significant lower incidence of HCV seroconversion in those who remained in treatment (AOR = 0.4, 95% CI 0–4.2) compared to those who had left (AOR = 1.0). Cessation of injecting at follow up was statistically significantly associated with continuing treatment (AOR = 0.1, 95% CI 0.1–0.2). This study is confirmed by the findings a Dutch prospective cohort study [47]. It found no statistically significant reduction in HCV incidence (chi-squared (χ^2^) test for trend P = 0.79) despite the provision of methadone programs, NEPs, free condom distribution and an information campaign. However the limitations of the study were that none of these variables where controlled for in the analysis.

Three separate observational studies evaluating the incidence of anti-HCV seroconversion amongst cohorts taking MMT did not demonstrate any statistically significant difference in incidence between those taking MMT and those not [18,43,44]. However, these studies only used univariate analysis. Additionally, only one study [44] reported the mean methadone doses that may affect the reduction in anti-HCV incidence. This may be important as some commentators have argued that under-dosing would reduce the effectiveness of MMT at reducing unsafe injecting behaviour [48,49]. Additionally, it has been argued that while users are likely to contract hepatitis C early in their injecting, they do not present to MMT services until later years, when they are more likely to have contracted HCV [49].

### Effect of behavioural programmes on HCV seroconversion

Behavioural interventions work within a framework of psychological theory. Such interventions can be delivered at the individual or group level. They seek to increase readiness to change by building trust and reducing resistance [50]. They seek to increase users self efficacy and their perceived discrepancy between their actual and ideal behaviour [51]. However, we were unable to identify any intervention studies evaluating the impact of behavioural programmes at reducing the incidence or prevalence of anti-HCV.

Three observational studies alluded to the effect of harm reduction programmes which included the effect of "outreach workers", "counsellors", or "advice" [47,52,53]. However none of these studies described the framework of psychological theory. Also none of the studies evaluated the interventions separately from other interventions such as NEPs, condom distribution or opiate maintenance therapy. Two studies [47,52] demonstrated a statistically significant reduction in HCV due to the overall programme. The other study noted a reduction in the prevalence of HIV after the introduction of preventive measures (condoms and safer injecting advice). Therefore it is not possible to draw definitive conclusions from these studies. It is plausible that "advice" or more structured behavioural interventions delivered alongside other harm reduction interventions does reduce the incidence of HCV but there is a need for further research to evaluate the effect of such interventions.

### Does bleach distribution reduce the risk of HCV?

Some commentators argue that training drug users to clean syringes effectively gives false assurance, reduces the validity of health advice to never share another person's injecting equipment and reduces the health policy imperative to ensure that sufficient needles are distributed [54]. However, recent qualitative research has shown that needle sharing is not a fixed behaviour, but is more likely when a user is withdrawing and has obtained drugs but does not have access to clean injecting equipment [55]. There appears to be limited evidence to inform best practice. One under-powered case control study nested within a prospective cohort study of 390 IDUs from five American cities reported a statistically non-significant reduction trend of lower anti-HCV seroconversion for those who used bleach all the time, compared to those who used it some of the time, to those who did not use it at all ()[56].

### Drug consumption rooms and hepatitis C

Drug consumption rooms (also known as supervised injecting rooms or medically supervised injecting centres) are legally sanctioned and supervised facilities designed to reduce the health and public order problems associated with illegal injection drug use [57]. Their purpose is to enable the consumption of drugs under hygienic, low-risk conditions. Trained health staff, while not physically helping users to inject illicit drugs, supervise injecting in order to avoid high-risk drug taking and to ensure hygienic practices. Part of their intended benefit is to reduce drug-related harm associated with transmission of blood-borne virus infections. Internationally, there has been a recent increase in the number countries operating drug consumption rooms though at the time of writing the UK does not have a legal framework sanctioning their provision. We were only able to find one evaluation of a drug consumption room that specifically studied anti-HCV conversion as an outcome. The evaluation was a time series analysis from an early evaluation of a drug consumption room in Australia. Whilst statistical analysis was reported in the paper, for the outcome of anti-HCV conversion descriptive data only was presented. Such data found no change in the incidence of notifications of hepatitis C infections among local users during the 18-month trial period, despite an increase in notifications from neighbouring areas [58]. The report acknowledges, however, that the low population prevalence of the infections in Australia may make it difficult to detect any statistically significant changes. A more recent report on drug consumption rooms concurred that few data are available regarding the impact of such centres on the incidence of drug-related infectious diseases [59]. It is plausible that these rooms can contribute to a reduced incidence of HCV given that numerous surveys show that high-risk users use such centres and report significant reductions in BBV risk behaviour [60-64].

## Discussion and conclusion

Reducing the incidence of HCV continues to present a considerable challenge. Recent UK based research conducted amongst injecting drug users documented an incidence rate of 41.8 per 100 person years for HCV and 3.4 per 100 person years for HIV [65]. Therefore in the absence of an immediate prospect of a vaccine against HCV [66], over-reliance should not be placed on any one harm and risk reduction intervention. Provision of clean needles and syringes are interventions for which there is an evidence base. Providing optimal dose opiate substitution therapy; drug consumption rooms as a hygienic place for those who engage in public injecting; behavioural interventions; and bleach and injecting paraphernalia distribution alongside needle and syringe distribution are all interventions that merit further expansion internationally supported by pragmatic research activity to contribute to the emerging evidence base. There is some evidence from the USA that sharing of "cookers" (usually the spoon or metal container used to prepare and heat drugs) presents a greater risk to the spread of HCV than the sharing of either cotton filters or water [67] though our review did not identify any studies evaluating the effects of paraphernalia distribution at reducing the incidence or prevalence of HCV.

One limitation of this review is that the comprehensive search was completed in 2002 to allow for submission and peer review by the WHO Health Evidence Network. Since that time some new literature has emerged in relation to prison based NEPs. An international review of prison based syringe exchange programmes published in 2003 reported that in small prisons with a high prevalence of injecting drug use, the introduction of NEPs led to a decrease in needle and syringe sharing over time whilst the prevalence of drug use decreased or remained stable. Whilst in one centre there were no new cases of HCV reported following the introduction of the NEP, there is a need for more epidemiological work quantifying the impact of NEPs in the prison setting upon HCV transmission.

**Figure 2:**
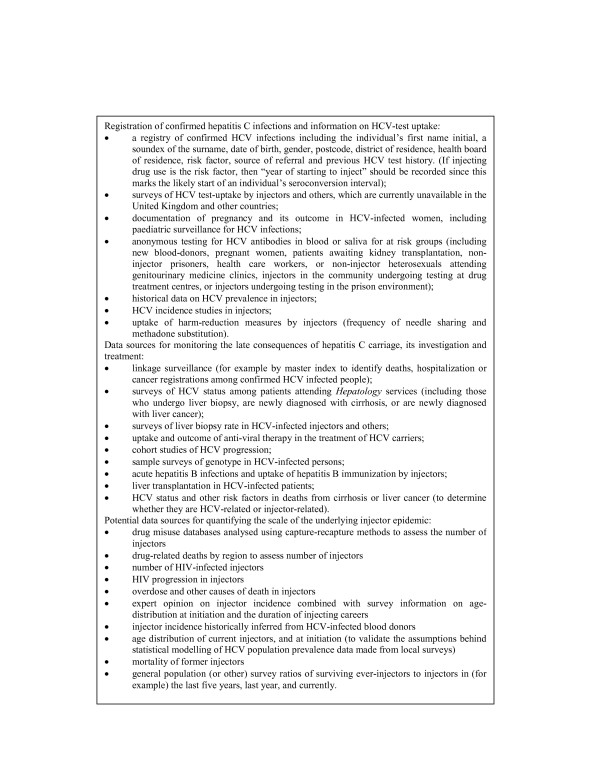
Proposed data sources for monitoring HCV incidence and prevalence.

Such work will require political will. Whilst internationally there has been minimal funding for pragmatic intervention trials in this field due to a lack of political will, the recently published UK Department of Health Hepatitis C Action Plan provides a window of opportunity to focus political, research and clinical resources upon the common goal of reducing the incidence of HCV [68,69]. However to inform resource allocation, policy makers will require improved data sources to monitor the societal health burden of the chronic sequelae of HCV infection. This need for improved data sources of HCV incidence and prevalence has been highlighted by some commentators [70]. Figure 2 highlights possible data sources in which they have proposed possible data sources for monitoring HCV incidence and prevalence amongst both injecting drug using and generic populations [70]. Monitoring trends amongst generic populations would have relevance to IDUs as it would provide ongoing data regarding the proportion of disease burden attributable to injecting drug use.

## Abbreviations

BBV – blood borne virus

ELISA – enzyme-linked immunosorbent assay

HCV – Hepatitis C

HIV – Human immunodeficiency virus

IDUs – injecting drug users

IVDUs – intravenous drug users

NEPs – needle exchange programmes

QALYs – quality adjusted life years

RNA – ribonucleic acid

WHO – World Health Organization

## Competing interests

The author(s) declare that they have no competing interests.

## Authors' contributions

Both authors preformed the literature search, read abstracts and determined include and exclude. CT was responsible for obtaining full text papers and NW read for further include, exclude and data extraction. NW prepared the first draft of the manuscript and both revised it accordingly. Both authors read and approved the final manuscript.

## Supplementary Material

Additional File 1 Summary of observational studies exploring the impact of primary prevention measures upon HCV prevalence and incidence among IDUs. The table summaries all relevant studies that have been included in the review Click here for file
