# Supplementary material for: A review of the evidence for the effectiveness of primary prevention interventions for Hepatitis C among injecting drug users
Source: Harm Reduct J. 2006 Sep 6;3:27. doi: 10.1186/1477-7517-3-27 (PMC1569828; doi:10.1186/1477-7517-3-27)
Supplement: Additional File 1 — Summary of observational studies exploring the impact of primary prevention measures upon HCV prevalence and incidence among IDUs. The table summaries all relevant studies that have been included in the review [file 1477-7517-3-27-S1.doc]

**Table 1 – Summary of observational studies exploring the impact of primary prevention measures upon HCV prevalence and incidence among IDUs**

| **Author, Year** | **Setting** | **Participants (number, age, gender, ethnicity & drug use)** | Methods **(Including inclusion/exclusion criteria & quality of methodology)** | **Duration of study** | **Outcomes** | **Results** |
| --- | --- | --- | --- | --- | --- | --- |
| Broers et al 1998 ^35^ | Methadone maintenance treatment clinic, Geneva, Switzerland | 706 drug users, 540 men, 166 women.  Average age at entry 27.0 years (range 17.4-48.4 years) | Prospective cohort study between 1988 and 1995 | Those in programme for 3 months compared with new clients | Effect of HIV health promotion interventions on HCV Incidence and prevalence for pre 1988 (drug use before HIV health promotion interventions); from 1988-1991 (mixed drug use and health promotion); 1991 onwards (drug use started post HIV health promotion interventions). Antibodies for HCV were assayed in 1989-1991 using a first generation HCV antibody ELISA system (Ortho-Diagnostics, Raritan, New Jersey, USA) and a second generation test from June 1991 (HCV EIA, Abbott). Stored sera were re-tested with the second test where available | Prevalence at entry into treatment declined dramatically over time for HCV. The prevalence of HCV among drug users entering treatment before 1988 was 91.6%, compared to 29.8% in those entering treatment after 1993. HCV incidence was 4.2% per person-year of follow up (95% CI 2.2-7.4) |
| Goldberg et al 1998 ^29^ | Regional Virus Laboratory, Glasgow, UK | 342 serum samples 1990 and 414 samples 1995 taken from IDUs presenting for HIV testing. Serum residues were stripped of patient information except for age and sex | Retrospective Longitudinal study | 2 cohorts – one tested in 1990 & one tested in 1995 | Prevalence of HCV in 1995 compared with 1990. Specimens reacting to ELISA (Ortho third generation, Chiron) were retested by a second ELISA (Sanofi Pasteur) hepatitis C test. Discrepant results tested by a recombinant immunoblot assay (RIBA third generation, Chiron) | Prevalence of anti-HCV fell significantly between 1990 and 1995 from 90% to 77% in IDUs of all ages (95% CI 73-81), from 92% to 29% in IDUs aged 15–19 (95% CI 1-56), and from 91% to 65% (95% CI 54-75) in IDUs aged 20–24. No significant reduction for those aged 25-29, 30-34 or those over 35. No significant differences between males and females in 1990 or 1995 |
| Goldberg et al 2001 ^30^ | Scottish Centre for Infection and Environmental Health, Glasgow | IDUs who had undergone named HIV testing HIV in Edinburgh and Glasgow were identified, linked to age band and gender information, and tested anonymously for HCV | Retrospective Longitudinal Study | Changes in anti-HCV prevalence in Glasgow over 1995-1997 and in Edinburgh for 1989-1990 and 1995-1997 | Changes in HCV prevalence. Residual sera specimens which tested reactive by a 3^rd^ generation ELISA assay (Ortho, Chiron) were retested with an ELISA assay (Sanofi Pasteur). Only specimens reacting to both tests deemed antibody positive | Significant decreases in anti-HCV prevalence in Edinburgh IDUs from 69% (95% CI 65-74) in 1989/90 to 13% (95% CI 8-21) in 1997 in those <25. Significant decrease in 25 years or over from 80% in 1989/90 (95% CI 76-83) to 54% in 1997 (95% CI 48-61). The χ² test for trend over 1989-97 showed the reducing trend to be more pronounced amongst those <25, (186.5, p<0.0001) than those 25 or over (54.6, p<0.0001). In Glasgow, a significant decrease, in prevalence from 91% (95% CI 85-95) to 43% in 1997 (95% CI 34-51) in those aged <25 (χ² test for trend 73.9, p<0.0001). For 25s and over, the decreasing trend was of borderline significance (3.7, p=0.06). Of both cities’ 17% of 15-19 year olds sampled during 1995-1997 were anti-HCV positive |
| Hagan et al 1995 ^37^ | Tacoma syringe exchange, USA | 28 IDUs (60.7% male, 82.1% white) were cases with acute HBV and 20 (70% male, 85% white) IDUs with acute HCV. Controls were 38 (50% male, 73.7% white) IDUs with no HBV markers and 26 (42.3% male, 73.1% white) with no HCV markers. 3 age groups, less than 25, 25-35 and 35 years or older | Case control study | 1991-1993 | Association between syringe exchange use/non use and hepatitis B and C in IDUs | After adjusting for demographic characteristics and duration of injecting drugs, non-use of the exchange associated with a seven fold greater risk of anti-HCV seroconversion (AOR = 7.3, 95% CI=1.6-32.8) |
| Hagan et al 1999 ^38^ | 6 drug treatment programs and from social service, corrections and drug-use assessment agencies Seattle, USA | 353 anti-HCV negative IDUs from a larger cohort of 2,728 IDUs recruited into an earlier study. Age ranged from 14, ethnic background was described as English or Spanish speaking. Recruitment by probabilistic sampling methods of every nth person as they entered the agency, or appeared on client lists. A control group was established | Prospective cohort | June 1994 and January 1996 | HCV incidence measured by 3^rd^ generation immunoassay (Abbott laboratories, Chicago, Illinois) | 70%) follow-u. No statistically significant differences between those lost to follow up and those retained in the study with respect to baseline characteristics 187/241 had injected during the follow-up period (mean of 408.9 days). From this cohort 39 IDUs seroconverted (a cumulative incidence rate of 20.8% per year). Relative to non-users, regular users had a slightly higher incidence (adjusted relative risk 1.31, 95% CI 0.79-2.19), which was lower than the incidence amongst sporadic users (adjusted RR 2.59 (CI: 0.79-8.5). Both effects statistically non-significant |
| Hernandez-Aguado et al 2001 ^53^ | 3 AIDS prevention & information centres, Spain | 5473 volunteers of which 3238 had an HCV test. Average age 27.4 years, 77.4% male | Prospective longitudinal study | 1990-1996 | Effect of HIV prevention measures upon the trends in prevalence of antibodies to HCV measured by a first generation EIA test during 1990-1991, a second generation EIA from 1992 onwards (Organon Teknika, Holland). A second confirmatory test (Recombinant Immunoblot Assay RIBA-2, Ortho Diagnostic systems, Raritan, New Jersey, USA) was done on the positive EIA tested serums | No statistically significant reduction in prevalence of HCV over the study period. 84.5% (1990-92 (RR=1); 84.1% 1993-94 (RR=0.99, 95% CI: 0.969-1.03); 87% 1995-96 (RR=1.03, 95% CI:0.99-0.07). Chi-squared test for trend NS (P=0.13) |
| Hutchinson et al 2002 ^31^ | Edinburgh, Glasgow, Tayside & Grampian, Scotland | Residual sera from IDUs who had undergone named HIV testing were tested anonymously for anti-HCV | Retrospective longitudinal study | Prevalence measured over 1989-2000 | Changes in anti-HCV prevalence since 1997 as tested by  an ELISA assay (3^rd^ generations Ortho, Chiron, or Abbott, Axsym). Reactive samples were retested by either a recombinant immunoblot assay (3^rd^ generation RIBA, Chiron) or Monolisa (Sanofi Pasteur) | No significant prevalence changes among those aged <25 during the late 1990s (Glasgow 1997-09/00: 43-41%; Lothian 1997-1999: 13-17%; Tayside 1997-1999: 45-35%; Grampian 1996-1999: 28-29%). The Tayside 1993-1999 reducing trend of anti-HCV prevalence was significant for the under 25s (57%-35%, χ² 3.9, p=0.05) and for those 25 or older (76% to 61%, χ² 6.6, p=0.01). Among those 25 years or older, significant decreases in prevalence were observed during the late 1990s in Glasgow [1997-9/00: 79% (95% CI 74-83) to 72% (95% CI 67-76)] and Lothian [1997-9: 54% (95% CI 48-61) to 45% (95% CI 39-51)] |
| Kapadia et al 2002 ^56^ | Recruitment through 6 sites in 5 US cities | 468 recruited. 390 persistently seronegative controls. 78 anti-HCV sero-converters. Age range 18-30 | Case control survey nested within a prospective cohort study | Data collected at baseline and 6 & 12 months | Effect of bleach disinfection of syringes on anti-HCV seroconversion | OR for anti-HCV seroconversion for participants who reported using bleach all the time = 0.35 (95% CI 0.08-1.62), OR anti-HCV seroconversion for those using bleach some of the time = 0.76 (95% CI 0.21-2.70) when compared to those not using bleach |
| Mansson et al 2000 ^34^ | Syringe/needle exchange program, Malmo, Sweden | 698 IVDUs, follow up possible for at least 6 months for 515. 76% male, median age 32 (range 20-58), ethnicity not stated. 70% amphetamine users, 16% heroin users, 14% both | Prospective cohort study | At least 6 months of the 2 year follow period | Effect of NEP upon incidence of HCV. Baseline sera samples tested for anti-HCV using licensed immunoassays. 1st generation screening used until April 1991, when replaced by second generation test. Seroconversion to anti-HCV confirmed by second or third generation recombinant immunoblot assay | HCV 26.3 seroconversions per 100 person years at risk. HCV seroconversion correlated with imprisonment during study (OR 2.2 95% CI 1.04-4.74), absence of drug free periods (OR 5.7 95% CI 1.44-22.3), and frequent needle, syringe exchange OR 1.31 95% CI 1.02-1.7) |
| MacDonald et al 2000 ^33^ | Needle and syringe programs (NSPs) in Australia. 21 NSPs in 1995, 20 NSPs in 1996 and 23 NSPs in 1997. | 4141 attendees at the NSPs (979 in 1995, 1463 in 1996 & 1699 in 1997). Age range 13-58, median age 28. 66% male | Repeated annual cross-sectional surveys for 1995, 1996 & 1997 | 1995-1997 | Prevalence of HCV as determined by capillary blood collected on blotting paper by finger prick and tested by third generation enzyme immuno-assay | Prevalence declined significantly from 63% in 1995 to 51% in 1996 and 50% in 1997 P<0.001. Remained significant when odds ratio adjusted for age, gender, duration of injecting, last drug injected, frequency of drug injection and health service contact (AOR 0.5, 95% CI, 0.4-0.7) |
| Patrick et al 2001 ^17^ | Vancouver, Canada | 1345 subjects (66% male, 34% female, median age 34 years, range 15-58, 60% white, 25% Aboriginal, 15% other) | Prospective cohort study | December 1996 to November 1999 | Effect of NEPs upon incidence of HCV as measured with a third-generation ELISA containing recombinant antigens (HCV 3.0, Ortho Diagnostics Systems, Rochester, NY)] at enrolment was 81.6% (95% CI 79.6%- 83.6%) | Multivariate analysis by Cox proportional hazards identified the following independent predictors for HCV seroconversion: female gender (adjusted hazard ratio 2.29 (95% CI 1.35-3.89), injection of cocaine alone or as a component of speedballs (adjusted hazard ratio 2.42 (95% CI 1.22-4.79) frequent injection (at least once per day,) adjusted hazard ratio 2.02, (95% CI 1.09-3.77) and frequent attendance at a needle exchange programme (at least once per week, adjusted hazard ratio 2.56 (95% CI 1.37-4.79) Insufficient power to determine a reducing trend in HCV incidence over the study period |
| Rezza et al 1996 ^45^ | Three drug treatment centres in Naples, Italy | 746 injecting heroin users. 263 IDUs were HCV negative at baseline and 40.3% were re-tested. Total follow up time 73.4 person years | Nested case control study within a prospective cohort study | Between 1991 and 1993 | Effect of MMT upon incidence of HCV, measured by EIA – Abbot laboratory test and confirmed by RIBA 2 test (Chiron, Corporation, Emeryville, California) | 21 individuals sero-converted, an incidence rate of 28.6/100 person years (95% CI 17.8-43.4). The AOR for “lack of methadone treatment” (in the 6 months prior to testing) was of borderline significance (2.9, 95% CI 0.9-9.7). |
| Selvey et al 1997 ^18^ | Methadone clinic in Brisbane, Australia. | 106 HCV negative clients (who had previously undergone testing) taking MMT identified from perusing drug treatment records of 319 users. No statistically significant differences in age, gender or duration of heroin use between those tested for HCV and those not. Median age 28 years (range 17 to 52), 61% male, 29% employed and median duration of heroin use was 7 years (range <1-27). The median duration since initial registration was 1 year (range 1 month to 5 years) | Prospective cohort study | November 1994 to March 1995 | Incidence of HCV for a cohort of IDUs taking methadone maintenance treatment at time of recruitment. Testing kits used to assess HCV status described as “the first negative test of all participants was performed by a second- or third-generation HCV ELISA, except in one instance, in which the result was later confirmed by third-generation testing” | Five seroconversions (14%) were recorded over 47 person-years, a seroconversion rate of 11 per 100 person-years (95% CI 2-20). Univariate analysis only conducted: time In methadone treatment was reported as “not associated with seroconversion”. However relative risk and supporting confidence intervals were not reported. Further univariate analysis described anti-HCV positivity associated with duration of heroin use at the time of the test (for duration 5-9 years RR 1.53, 95% CI 1.16-2.01, P<0.01 and 10+ years RR 1.96 95% CI 1.55-2.48, P<0.01) and being female for those cases whose duration was less than five years (RR 1.71 95% CI 1.08 to 2.71, P<0.05) |
| Smyth et al 1999 ^52^ | Addiction treatment centre in Dublin, Ireland | 353 injecting drug users with an injecting history of less than 24 months. Heroin 78%, morphine sulphate 21%, benzodiazepines 1%. Age range or mean age not stated. 68% male. Ethnicity not stated | Repeated cross-sectional surveys | New attenders between July 1993 & December 1996 | Effect of “expanded harm reduction programme” (needle exchange programme plus an increase in outreach workers and addiction counsellors) on prevalence of anti-HCV (confirmed by third generation enzyme linked immunosorbent assay) for those who injected pre August 1993, August 1993-July 1994 and those commencing after July 1994 | Statistically significant reduction in those commencing injecting post 1994 (AOR 0.43, CI 0.27-0.67, P value < 0.001) compared to those commencing injecting pre-1994. Statistically significant reduction in prevalence in those injecting less than 13 months (adjusted OR 1.0) compared to those injecting > 13 months (adjusted OR 1.76, CI 1.10-2.80, P=0.017). Statistically significant reduction in anti-HCV prevalence over time for those injecting less than 13 months (P< 0.003), but not those injecting > 13 months (P=0.33) – confidence intervals not reported |
| Somaini et al 2000 ^36^ | Four clinics offering opiate substitution in Zurich, Switzerland | 603 drug users, mean age 30.7 years (SD 6.2). 62% male. Ethnicity not stated. All but one had a history of heroin use and 80% reported a history of cocaine use. 75% gave a history of injecting drug use | Cross sectional study | 6 months from July 1997-January 1998 | Associations between NEPs and HCV prevalence. Exact serological testing procedures not stated and differed in the clinics, although | Protective effect in “the order of 80% for those starting to IDU after 1991 as opposed to those starting before 1987”. Statistical analysis not presented. Data presented in graphical form. |
| Taylor et al 2000 ^32^ | Glasgow, Scotland | 1949 saliva specimens from injectors from both in treatment and out of treatment settings. Age range 16-49, median 26 and median length injecting career 7.4 years. 72% male | Prospective study of annual cross sectional survey data between 1990-1994 and 1996. | 1990-1996 | Effect of NEPs on the annual prevalence of antibodies to HCV among IDUs as determined by a modified ELISA assay (Monolisa Anti-HCV-Sanofi Pasteur, France) to detect antibodies in saliva | 1189/1949 (61%) were anti HCV positive (95% CI-59%-63%). Prevalence rates per year ranged from the highest of 67% in 1990 (95% CI 62%-72%) to the lowest of 56% in 1996 (95% CI 49%-63%). Overall estimated seroprevalence of anti HCV was 72%. In multiple regression model, length of injecting career, year commenced injecting, number of times in prison since injecting & place of residence were significantly more likely to test positive. Those who began injecting after introduction of needle and syringe exchange were significantly less likely to test HCV antibody positive than those who started before |
| Thiede et al 2000 ^46^ | 4 methadone treatment centres, Washington, USA | 716 participants (83% participation rate from a systematically selected sample of 999). One year follow up rate was 84% Age > 14 years; median age 38 years. 51% male, 77% white. 74% started injecting 10 or more years before study enrolment | Prospective cohort study of incidence | Between October 1994 and January 1998. Baseline data collected and follow-up data at 12 months | Effect of methadone maintenance treatment on the incidence of HCV for those who “left methadone treatment”, “disrupted methadone treatment” or “continued treatment for the follow up period.” Testing for anti-HCV was by a third generation enzyme immunoassay (EIA; Abbott Laboratories) with repeat testing to confirm positive results | Statistically non significant reduction in HCV seroconversion for those who remained in treatment AOR 0.4 (95% CI 0-4.2) |
| Van Ameijden et al 1993 ^47^ | Low-threshold methadone programs and an STD clinic for drug using prostitutes, Amsterdam, The Netherlands | 305 heterosexual drug users who had an intake visit and at least 1 follow up visit between December 1985 and September 1989. 46% males, 69% Dutch, 21% German. Mean age 31.2 years (SD 5.8) for males and 27.2 years for females (SD 5.2). At intake 88% had ever injected | Observational cohort study with 4 monthly follow up with standardised questionnaire, medical examination and blood test for HBC, HCV & HIV | December 1985-September 1989 | Effect of “NEPs, information campaign, free distribution of condoms and methadone maintenance upon the incidence of HCV. Antibodies for HCV were assayed by a first generation HCV antibody ELISA system (Ortho Diagnostics, Raritan, NJ) | No statistically significant reduction in annual incidence rate/100 person years over the four year study period (1986: 16.9; 1987: 4.0; 1988: 12.5; 1989: 11.2) chi-squared test for trend, P=0.79) |

Note: some studies report incidence/prevalence for HBV and HIV. Only the data for HCV is presented in this table
